# The ABCs of DKA: Development and Validation of a Computer-Based Simulator and Scoring System

**DOI:** 10.1007/s11606-015-3273-y

**Published:** 2015-07-15

**Authors:** Catherine H. Y. Yu, Sharon Straus, Ryan Brydges PhD

**Affiliations:** 1 St. Michael’s Hospital, Toronto, ON USA; 2 Department of Medicine, University of Toronto, Toronto, ON USA

**Keywords:** medical education, assessment/evaluation, medical education, clinical skills training, medical education, computer/web-based training, medical education, instructional design, medical education, simulation

## Abstract

**Background:**

Clinical management of diabetic ketoacidosis (DKA) continues to be suboptimal; simulation-based training may bridge this gap and is particularly applicable to teaching DKA management skills given it enables learning of basic knowledge, as well as clinical reasoning and patient management skills.

**Objectives:**

1) To develop, test, and refine a computer-based simulator of DKA management; 2) to collect validity evidence, according to National Standard’s validity framework; and 3) to judge whether the simulator scoring system is an appropriate measure of DKA management skills of undergraduate and postgraduate medical trainees.

**Design:**

After developing the DKA simulator, we completed usability testing to optimize its functionality. We then conducted a preliminary validation of the scoring system for measuring trainees’ DKA management skills.

**Participants:**

We recruited year 1 and year 3 medical students, year 2 postgraduate trainees, and endocrinologists (*n* = 75); each completed a simulator run, and we collected their simulator-computed scores.

**Main Measures:**

We collected validity evidence related to content, internal structure, relations with other variables, and consequences.

**Key Results:**

Our simulator consists of six cases highlighting DKA management priorities. Real-time progression of each case includes interactive order entry, laboratory and clinical data, and individualised feedback. Usability assessment identified issues with clarity of system status, user control, efficiency of use, and error prevention. Regarding validity evidence, Cronbach’s α was 0.795 for the seven subscales indicating favorable internal structure evidence. Participants’ scores showed a significant effect of training level (p < 0.001). Scores also correlated with the number of DKA patients they reported treating, weeks on Medicine rotation, and comfort with managing DKA. A score on the simulation exercise of 75 % had a sensitivity and specificity of 94.7 % and 51.8%, respectively, for delineating between expert staff physicians and trainees.

**Conclusions:**

We demonstrate how a simulator and scoring system can be developed, tested, and refined to determine its quality for use as an assessment modality. Our evidence suggests that it can be used for formative assessment of trainees’ DKA management skills.

## BACKGROUND

Diabetic ketoacidosis (DKA) accounts for an estimated 115,000 hospital discharges per year in the USA.^1^ Clinical management is suboptimal; in a single-centre chart audit of 55 patients admitted with DKA to a large teaching hospital, the mean time to insulin initiation (a key component of therapy) was 207 min, and 75 % were placed on an inappropriate hyperglycemia protocol that did not address the other metabolic derangements of DKA.^2^

DKA is a medical emergency necessitating hourly assessment of a myriad of dynamic clinical parameters, resulting in numerous critical decision-making points, which are further complicated by the complex interplay between management actions.^3^ While clinical knowledge is necessary, clinical reasoning and management skills are critical for successful patient management. One before-after study examined the effect of resident education on DKA knowledge^4^. Fifty-one residents undertook a web-based test consisting of 12 multiple-choice questions before and 6 months after the intervention. In addition to receiving test feedback and links to further reading, they attended two 1-hour didactic lectures and case-based discussion. The authors reported no change in resident knowledge between the two time points. How best to improve residents’ clinical reasoning and management skills related to DKA has yet to be studied fully.

In contrast to passive delivery of content (i.e., didactic lectures), research has shown that trainees acquire skills and develop expertise through deliberate practice. Ericsson^5^^,^ 6 describes deliberate practice as a set of “…activities that have been found most effective in improving performance,” consisting of nine elements: highly motivated learners, well-defined learning objectives, appropriate levels of difficulty, focused repetitive practice, reliable measurements, informative feedback, monitoring and error correction, evaluation and performance, and advancement to the next task.^7^

A meta-analysis comparing simulation-based training in which trainees followed deliberate practice principles to traditional clinical medical education found 14 studies (6 randomized trials, 3 cohort, 1 case-control, and 4 pre-post studies), which addressed procedural, auscultation, and life support skills in medical students and residents.^7^ All studies favored simulation-based training with deliberate practice over traditional education, with an overall effect size correlation of 0.71 (95 % CI 0.65–0.76, *p* < 0.001). Thus, deliberate practice has strong potential as a framework for designing the training and assessment of clinical skills, including medical students’ and residents’ DKA management skills.

These previous studies on deliberate practice have not clarified which of the nine elements are most responsible for the observed performance improvements. In order to optimize the effectiveness of educational interventions employing deliberate practice, a rigorous understanding of its key elements and the contribution of each is central. For example, Pusic et al. have demonstrated that repetitive practice, one of the key elements of deliberate practice, is essential for trainees to develop expertise.^8^ In a prospective cross-sectional study, 18 pediatric residents were asked to classify whether 234 cases of ankle radiographs were normal or abnormal. Learning was greatest between cases 21 to 50, highlighting the importance of repetitive practice in gaining expertise. Given the high number of repetitions required to gain expertise, Pusic et al. suggest that computer simulation is an ideal medium for tracking the development of deliberate practice and for clarifying which of its nine elements are most useful.^9^

Two of the key elements of deliberate practice are that informative feedback be provided from educational sources and that assessment scores are available to produce a mastery standard.^7^ Thus, before a simulator can be used as a medium for deliberate practice, it must have a robust scoring system for which favorable validity evidence exists. Recently, Cook et al. conducted another review of the simulation literature specifically looking for validity evidence and found a paucity of reports.^10^^,^ 11In particular, they noted little use of validity frameworks, which have been the gold standard approach in the fields of psychology and education since 1999.^12^

## OBJECTIVES

We aimed to develop a computer-based DKA simulator for medical training that included a robust scoring system. We collected validity evidence in order to judge whether the scores are appropriate measures of undergraduate and postgraduate medical trainees’ DKA management skills for both formative (e.g., identify students who require additional training) and summative (e.g., identify students who are competent) purposes. We chose to use the National Standards framework, which emphasises the collection of five sources of validity evidence, including content, response process, internal structure, relations with other variables, and consequences.^12^^,^ 13

## DESIGN

### Overview

First, we developed the DKA simulator, which relied on expert review of content. Next, we conducted usability testing of the simulator, which led to refinement of its content and functionality. We then developed the simulator scoring system and assessed our hypothesis that the in-built scoring system would produce favorable validity evidence demonstrating it is an appropriate measure of trainees’ DKA management skills.

### Aim 1: Simulator Development and Refinement

#### Simulator Development

##### Content

The principal investigator (CY) identified key principles regarding DKA management in accordance with the Canadian Diabetes Association 2013 Clinical Practice Guidelines (CDA CPG)^3^ and incorporated those principles into clinical scenarios. In addition, she created linear equations that were modeled to simulate real-life parameters, such as vital signs and laboratory abnormalities. Six scenarios were designed to reflect the variety of presentations and management challenges (e.g., DKA with concurrent respiratory alkalosis; Appendix 1). Real-time progression of the case scenario included patient clips, interactive order entry, and presentation of laboratory and clinical data.

##### Format

In keeping with best practices for the instructional design of simulation activities, we designed the simulator to include interactivity, individualized learning, preset action categories, feedback, repetitive practice with varying levels of difficulty, and contrasting cases.^14^^–^ 18 Specifically, learning was individualized based on the user’s actions, for example, if they failed to administer potassium, the “patient’s” serum potassium would fall and the user would receive specific feedback regarding aggressive potassium replacement. The simulator consisted of preset action categories, including items under clinical assessment, investigations, management, and nursing. Users received feedback based on their actions throughout and upon completion of the simulation, consisting of “Helpful Hints,” as well as a summary report indicating their performance in each management category and additional reading. For example, if they did not order an arterial blood gas, they were prompted to do so and given the rationale for ordering it. Finally, the simulator included six contrasting clinical scenarios with varying difficulty (for example, an older adult in hyperosmolar hyperglycemic state; Appendix 1). We also implemented elements of deliberate practice in our design: well-defined learning objectives or tasks; appropriate level of difficulty; informative feedback from educational sources; focused, repetitive practice; rigorous, reliable measurements; and monitoring, error correction, and more deliberate practice.^7^

##### Programming and platforms of delivery

The extensive programming required for the complex interactions between the simulated patient’s parameters and the learner’s actions was completed by a programmer with the LAMP stack (Linux Ubuntu Distro, Apache 2.0, MySQL 5.0 and PHP 5.3), CodeIgniter as the Model-Viewer-Controller framework, jQuery for front end logic, and the HTML5 Boiler Template and Modernizr to expedite development for cross-browser compliance. The computer-based program was delivered over the Internet and run on standard web browsers. Iterative design, refinement, and quality assurance occurred over a 12-month period.

#### Expert Review of Content

We invited four clinical experts (one endocrinologist, one intensivist, one general internist, and one emergency physician) in active clinical practice (>50 % of time performing clinical work) with frequent exposure to DKA, through convenience sampling. Each independently completed each scenario to assess the accuracy and realism of the content and was asked to complete a questionnaire assessing inaccuracies (Appendix 2). The questionnaire was developed by CY and reviewed by SES. In addition, CY took field notes of their comments as the expert ran through each simulator case (although this was not a formal think-aloud protocol).

#### Usability Testing

The simulator underwent heuristic evaluation by a human factors engineer (SJ). Heuristic evaluation is conducted by usability experts, who review the product using a set of validated usability heuristics as guidelines, following the methodology defined by Nielsen.^19^ Usability issues were categorized by severity into minor, moderate, major, or catastrophic.

#### Simulator Refinement

Based on recommendations from the expert content review and usability phases of our process, the prototype was modified through an iterative process of design and evaluation. Specific changes are described in the Results section.

### Aim 2: Collecting Validity Evidence for the DKA Simulator Scoring System

#### Development of the Scoring System

We modeled our simulator scoring system from those in the literature.^20^ The seven priorities in DKA management^3^ comprise the seven domains of the scoring system, which are (1) potassium deficiency, (2) volume depletion and fluid replacement, (3) acidosis, (4) hyperglycemia, (5) precipitating cause, (6) organization of care (e.g., communication with nurse), and (7) monitoring of patient. This comprised a total of 18 performance items (Appendix 3). For each performance item, the simulator tabulated percentage of correct actions and identified critical errors performed. The simulator then calculated a 3-point scoring scale per item^20^ (Appendix 3), resulting in a final numerical score ranging from 18 to 54, where 18 represented unacceptable performance in all performance items and 54 represented acceptable performance in all performance items.

#### Collection of Validity Evidence

##### Setting

We conducted the validation phase at a large urban academic health sciences center.

##### Participants

We recruited individuals with varying levels of expertise in DKA management [undergraduate medical students in year 1 (MS1) with limited knowledge and expertise, undergraduate medical students in year 3 (MS3), postgraduate trainees in year 2 of internal medicine residency (PGY2), and staff endocrinologists with extensive knowledge and expertise]. We asked all participants to complete the simulator after viewing a tutorial and completing one practice run to familiarize them with the simulator; hints were not given during the practice run. Sample size could not be estimated based on previous data, as this was a new scoring system. However, we expected a large effect size (given the wide variation in expertise of the groups) and estimated that to achieve a power of 0.80 with an alpha of 0.05, a total sample size of 66 participants would be required.^21^

##### Main Measures

As outlined in Messick’s^12^ original work and itemized for medical education researchers,^10^ validity evidence can be organized into five categories. We note that it is not necessary (or usually possible) to collect all sources of validity evidence in a single study.^10^ Consequently, our methods emphasized assessment of validity related to content, internal structure, relations with other variables, and consequences (Table 1). A recent article provided an organizing framework that we used to choose which data elements to collect^10^ (Table 1).

**Table 1.** Data elements collected for each category of validity evidence

Validity evidence	Description	Data elements collected
Content evidence	Match between assessment content and measured constructs	We adapted a pre-existing framework^20^ because of its applicability to this content area, as well as CDA CPG,^3^ to develop the scoring method, and subsequently had content and education experts review the scoring system
Internal structure	Relations of the assessment items with the overarching construct	We assessed the internal consistency of the seven subscales composing the scoring system using Cronbach's alpha. We also conducted an exploratory factor analysis to explore the relationships between the subscales to further contribute to internal structure evidence
Relations with other variables	Statistical associations between assessment scores and other measures	We compared mean scores for each trainee group using a one-way analysis of variance (ANOVA), with group membership as the between-subjects variable. We also examined correlations between the simulator score and participants’ characteristics [previous exposure to DKA, time spent on a Medicine rotation (medical students only), self-reported comfort, age and gender] using Pearson’s correlation coefficient
Consequences	Impacts of the assessment and the related decisions about trainees	We determined a pass/fail cut point for the scoring system using receiver-operating characteristic (ROC) analysis in the Statistical Product and Service Solutions (SPSS version 20) software package to determine the threshold for discriminating between staff endocrinologists and trainees (i.e., MS1, MS3, and PGY-2) on the DKA simulator.^22^ We aimed to identify this cut point for both formative (e.g., identify students who require additional training) and summative (e.g., identify students who are competent) purposes. We plotted the ROC curve to display the relationship between sensitivity and specificity and to identify the optimal threshold for discriminating between the levels of expertise (i.e., cutoff score). To provide a measure of the accuracy of the threshold, we calculated the area under the ROC curve, as well as the Youden index (J), the sum of sensitivity and specificity minus one

##### Ethical review

We obtained approval from the ethical review board of the involved institution. Informed consent was obtained from all participants.

## KEY RESULTS

### Aim 1: Simulator Development and Refinement

#### Simulator Development

We depict simulator functionality and representative screenshots in the Appendices (Appendix 4: Simulator tutorial; Appendix 5a-i: Screen shots of simulator).

#### Expert Review of Content

Clinical experts thought that the simulator was reflective of real-life management of DKA. However, they felt that other medical care (for example, management of congestive heart failure) was neglected to focus on DKA. A summary of their comments is provided in Appendix 2.

#### Usability Testing

No critical issues were identified. However, some usability issues were rated as ‘major’ or ‘moderate.’ For example, the purpose of “Notes to self” was not clear. Most of these were thought to be adequately addressed through training or by additional explanatory text (Table 2).

**Table 2.** Simulator refinement based on heuristic evaluation

Section	Problem category	Problem	Action
Results	Consistency with standards	When new information is available (e.g., new results arrive), this is indicated by changing the title font color from green to red. However, red and green are typically used to indicate abnormal/dangerous and normal states. This can cause unnecessary confusion	Change to black font instead of green, and indicate new results using a bold version of the same font, as is standard in email applications. Add the number of results in parentheses, e.g., Results (2 new)
Notes to self	Visibility of system status	It is not clear whether this section has a real-life equivalent in the clinical setting, other than informal notes to self, which could be done using any method to which the user is accustomed. Also, its contribution to the total score is unclear	Change title to “Medical notes” Add a brief text (e.g., currently in the blank text area) in gray (that disappears if they try to type in it): “Use this area to type in medical notes (optional)”
Flow sheet	Visibility of system status	Although mentioned in the training presentation, users may forget that this section is optional and that it needs to be filled in manually	Add a brief text below the title: “Use this flowsheet as you would in a real-life clinical scenario”
Investigations	Visibility of system status	After a test is ordered by clicking ‘Submit,’ the ‘Submit’ button is grayed out. There is no feedback to the user on this section on the screen	Add a popup that can be disabled by the user: “[test] has been ordered and will be available in [x time]”
Flowsheet	User control and freedom; flexibility and efficiency of use	No scrollbar (using Firefox v. 14.0.1) when adding additional rows in the flowsheet (see Fig. 1). Also, the columns do not fit the width of the page. At least one column heading (starting with “A”) is cutoff	Check browser compatibility
Heading	Consistency with standards	It is not clear that the text “+5,” “+10,” etc., is clickable	Replace the text with buttons (which are clearly clickable)
Exit–heading	Error prevention	When “Exit” is clicked, no feedback or warning is provided, and all data appear to be immediately lost. The user may expect a message that would explain what would happen	Adding a message, as in when “end simulation” is clicked, which would clearly state that the simulation will end immediately, without providing the score
Physical examination	Error prevention	The purpose of the text “Talk to nurse to order vital signs” under “Physical examination” is confusing. Some users may try to click on the text, as it appears under a section where the results are to be ordered	Transforming the text “Talk to the nurse” into an active link. If that is not possible, add additional instructions (e.g., “click the “Talk to Nurse” button on the left to order vital signs”)
Communication section	Visibility of system status; internal consistency	Communication section notifies the user when the results will be available at the time of ordering, but does not notify the user when the results become available	Communication section should also list when the results become available (same message as pop-up)
Heading	Functional error	BP information was not updated in the top part of the screen, but it was updated in the “nurse” window. Compare with HR value, which was updated in the heading	Correct bug

#### Simulator Refinement

Based on recommendations from expert content review and heuristic evaluation, the prototype was modified by team members through an iterative process of design; refinements are indicated in Table 2. For example, we renamed “Notes to self” as “Medical Notes” and added a brief text below stating its purpose.

### Aim 2: Collecting Validity Evidence for DKA Simulator Scoring System

Eighty-one participants were recruited to the study (Table 3). Sixty-eight participants (91 %) reported using other forms of information technology for medical-related learning (primarily online resources such as Up-to-date); of these, 0 participants reported previous exposure to simulation-based learning. On inspection of the data distribution, we identified six participants with scores greater than two standard deviations from the mean. Five of these outliers spent less than 60 seconds on the simulator, indicating that they did not complete the patient case and the sixth performed very poorly. We chose to eliminate all of these individuals from further analyses, leaving us with 75 participants in total.

**Table 3.** Participant characteristics

	Medical students - year 1, *n* (%)	Medical students - year 3, *n* (%)	Postgraduate trainees - year 2, *n* (%)	Staff endocrinologist, *n* (%)	Total (*n* = 75)
N	18	21	17	19	75
Age group					
21–30 years old	20 (100 %)	20 (95 %)	16 (94 %)	0	55 (72 %)
31–40 years old	0	1 (5 %)	1 (6 %)	14 (74 %)	16 (21 %)
41–50 years old	0	0	0	3 (16 %)	3 (4 %)
51–60 years old	0	0	0	1 (5 %)	1 (1 %)
>60 years old	0	0	0	1 (5 %)	1 (1 %)
Male gender	11 (58 %)	10 (48 %)	6 (35 %)	9 (47 %)	36 (47 %)
English as first language	14 (74 %0	18 (86 %)	13 (76 %)	16 (84 %)	61 (80 %)
Weeks on general internal medicine (months for postgraduate trainees - year 2)					
0	18 (100 %)	8 (38 %)*	0	N/A	N/A
1	0	1 (5 %)	1 (6 %)	N/A	N/A
2	0	0	5 (29 %)	N/A	N/A
3	0	0	3 (18 %)	N/A	N/A
4	0	0	6 (35 %)	N/A	N/A
5	0	1 (5 %)	2 (12 %)	N/A	N/A
>5	0	9 (43 %)	0	N/A	N/A
Years in practice					
<5 years	N/A	N/A	N/A	6 (32 %)	N/A
5–10 years	N/A	N/A	N/A	8 (42 %)	N/A
11–15 years	N/A	N/A	N/A	2 (11 %)	N/A
15–20 years	N/A	N/A	N/A	1 (5 %)	N/A
>20 years	N/A	N/A	N/A	2 (11 %)	N/A
Comfort with managing diabetes					
Very comfortable	0	0	2 (12 %)	15 (79 %)	17 (22 %)
Comfortable	0	1 (5 %)	10 (59 %)	4 (21 %)	15 (20 %)
Neutral	1 (5 %)	8 (38 %)	4 (24 %)	0	13 (17 %)
Uncomfortable	5 (28 %)	8 (38 %)	1 (6 %)	0	15 (20 %)
Very uncomfortable	12 (67 %)	4 (19 %)	0	0	16 (21 %)
Number of DKA patients treated					
0 patients	18 (100 %)	18 (86 %)	1 (6 %)	0	50 %
1–5 patients	0	3 (14 %)	12 (71 %)	1 (5 %)	16 (21 %)
6–10 patients	0	0	3 (18 %)	1 (5 %)	4 (5 %)
11–15 patients	0	0	0	4 (21 %)	4 (5 %)
16–20 patients	0	0	1 (6 %)	1 (5 %)	2 (3 %)
>20 patients	0	0	0	12 (63 %)	12 (16 %)

*Two participants did not respond

**Figure 1.**
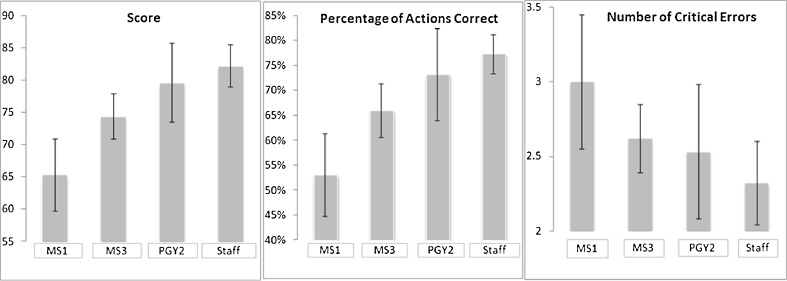
Mean score, percentage of actions correct, and number of critical errors by level of training. Error bars indicate standard deviation; undergraduate medical students in year 1 (MS1) with limited knowledge and expertise, undergraduate medical students in year 3 (MS3), postgraduate trainees in year 2 of internal medicine residency (PGY2), and staff endocrinologists.

1. Content: We based our scoring system on a pre-existing framework,^20^ the CDA CPG,^3^ as well as expert review by content and education experts reported above.
2. Internal structure: For the seven subscales, Cronbach’s α was 0.795, indicating adequate internal consistency. The exploratory factor analysis revealed that the Kaiser-Meyer-Olkin value was 0.25, which suggests our sample size was inadequate for conducting such an analysis (the value should be >0.50).
3. Relations with other variables: According to our ANOVA, the mean overall simulator score showed a significant group difference (F (3, 71) = 11.2, *p* < 0.001). Post-hoc analyses using Tukey’s HSD revealed the source of the difference was that the MS1 group scored significantly lower than all other groups (*p* < 0.02). The other groups’ scores did not differ significantly (Figs. 1 and 2). Our correlation data suggested that self-reported comfort with managing DKA correlated with the simulator score (*r* = 0.55, *p* < 0.001), as did the medical students’ self-reported number of weeks on GIM rotation (*r* = 0.40, *p* < 0.014). Similarly, across all groups, our nonparametric variables of age and number of DKA patients treated correlated with score (*p* = 0.022 and *p* < 0.001, respectively). There was no correlation of score with residents’ self-reported number of months on GIM rotation or gender.

**Figure 2.**
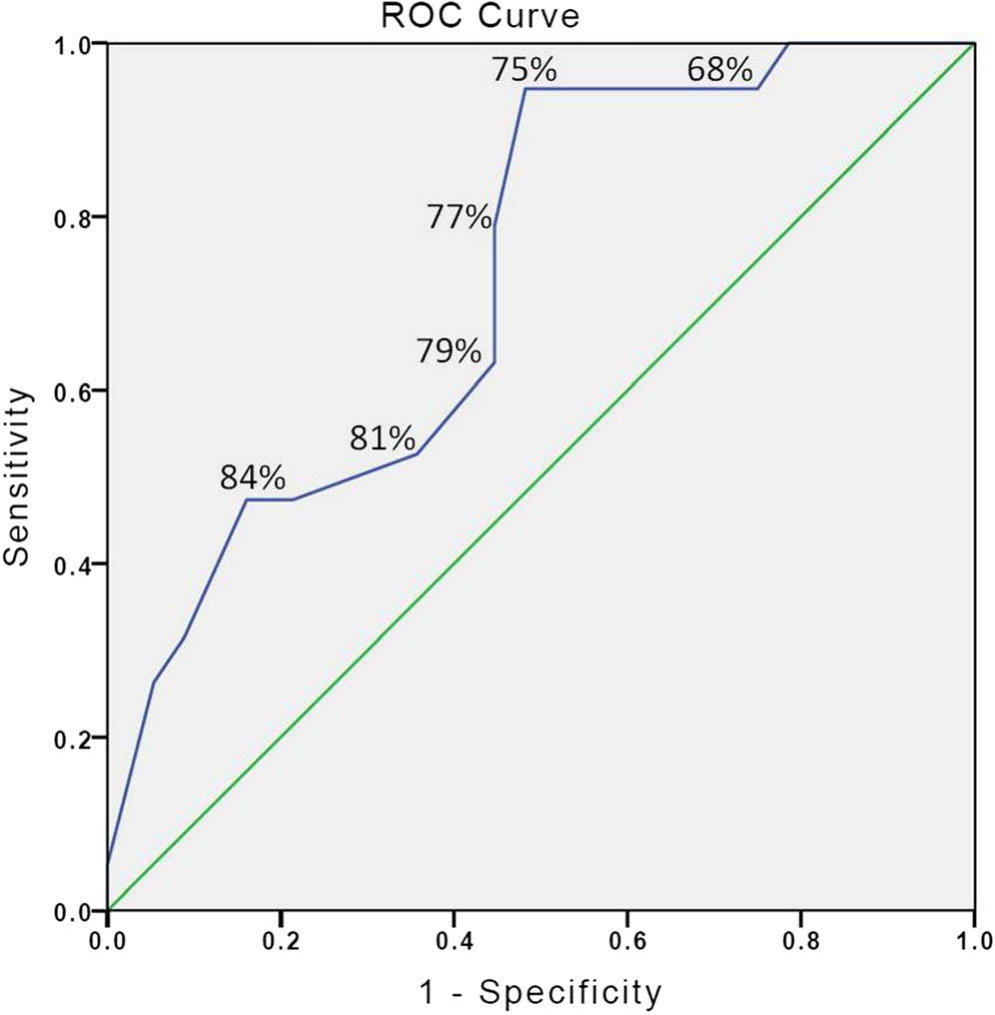
Receiver-operating characteristic curves for discriminating between expert and non-expert on the basis of score. The number indicated for each point is the score applied as a cut point value.
4. Consequences: We generated a receiver-operating characteristic (ROC) curve to define a simulator cutoff (“pass-fail”) score that would delineate a threshold between practicing physicians (considered ‘experts’) and trainees. Using the data from the curve, we calculated the Youden index (sum of sensitivity and specificity minus one) in order to identify the optimal cutoff score. We found that the largest value (0.47) occurred at a simulator score of 75 % (sensitivity of 94.7 %, specificity 51.8 %), demonstrating that a score of 75 % has high sensitivity (cutoff scoreable to identify 94.7 % of practicing physicians) but low specificity (cutoff scoreable to exclude 48.2 % of trainees). The area under the curve was fair at 0.73 ± 0.06 (95 % confidence interval: 0.61–0.85, *p* = 0.003).

## CONCLUSION

We integrated guideline-based content and expert input, evidence-based instructional design strategies, and principles of user-centered design to develop an easy-to-use, engaging, and realistic computer-based DKA management simulator. We also evaluated validity evidence and judged the value of the evidence using two elements of deliberate practice: that informative feedback is provided from educational sources and that assessment scores are available to produce a mastery standard.^7^ Our judgment of the validity evidence is that it is mostly favorable for using the DKA simulator as a formative method for assessing trainees’ skill in DKA management. However, the data do not substantiate using the simulator for summative purposes: although performance of junior medical students differed from other groups, the low specificity of our cut point score suggests the scoring system is not yet sensitive to subtle DKA management performance differences between senior medical students and residents.

### Current Validity Argument for Use of the DKA Simulator/Criteria for Effective Assessment

For a test to provide effective *formative* assessment for the learner, it should provide specific and actionable feedback, be integrated into the learning experience, and be timely and ongoing.^23^ Our DKA simulator provides feedback based on the learner’s actions and suggests correct management actions throughout the simulation and upon completion. Based on our content and relations with other variables' evidence, the simulator appears able to assess and differentiate a learner’s ability to identify and prioritize management options. Further research is needed, however, to ensure the feedback provided leads to performance improvements during prolonged periods of deliberate practice.

For a test to provide effective *summative* feedback for the learner and educator, it must consist of high-quality test material, a systematic standard-setting process, and secure administration as well as demonstration of validity, consistency, and equivalence.^23^ We created high-quality test material that was securely administrated and initiated a systematic standard setting process. However, although our collection of internal structure evidence demonstrated good internal consistency, our collection of consequence evidence, specifically the psychometric properties of the cutoff score, was not sufficiently strong to support its use for summative purposes; although sensitivity was high at 94.7 %, specificity was low at 51.8 %, thus not permitting accurate prediction of expertise. In addition, we have not yet assessed test-re-test reliability or equivalence (i.e., whether the same assessment yields equivalent scores or decisions when administered across different institutions or cycles of testing). In order to build upon a validity argument wherein the simulator score can be used to predict practice-ready competence in DKA management, additional consequence evidence such as evaluation with the actual pass rate (e.g., on objective structured clinical examination) will need to be collected.

### Strengths and Limitations

Strengths of our simulator include its systematic development. User-reported limitations include its focus on DKA management, to the exclusion of other medical conditions; this was deemed an acceptable compromise given the intended focus of the simulator. A study strength includes our collection of multiple sources of validity evidence, which resulted in a more balanced assessment of the validity of our scoring system. Unlike previous studies in the literature,^10^^,^ 11 we collected not only evidence for relations with other variables, but also evidence for content, internal structure, and consequences. We believe this study serves as an example in moving the field of validation research methods forward in the domain of simulation-based medical education and assessment.

### Next Steps

The current study is the first in a program of study that ultimately is aimed at impacting translational outcomes such as patient care practices, better patient outcomes, and collateral educational effects.^24^ For example, integration of the simulator into the medical curricula may improve resident knowledge and skills, the mean time to insulin initiation, prevalence of life-threatening hypokalemia, adequate fluid resuscitation, and subsequently patient morbidity and length of stay. Next steps of this research program are to explore further refinements to the scoring algorithm, how to most effectively implement the simulator in a curriculum, such as the optimal setting (for example, on-site invigilation by a coach versus self-study), and the optimal dose (for example, set number of case repetitions versus self-selected number of case repetitions). In addition, the simulator can be used to collect participant responses to clinical cues, which may be used to better understand the mechanism by which simulator cases can improve skills. Furthermore, the impact on clinical reasoning and the time course for these changes can be explored. Thus, computer-based simulation offers opportunities to improve trainee skill and to better understand how trainees learn.

Using the principles of deliberate practice and incorporating evidence-based instructional features, we developed a computer-based DKA management simulator. We subsequently collected an array of validity evidence for the scoring system including evidence on content, internal structure, relations with other variables, and consequences. Our next steps are to explore refinement of the scoring system and integration of the DKA simulator into medical education; pending these findings, the simulator will be refined and made available to the broader medical education audience.

## Figures and Tables

**Table 5.**
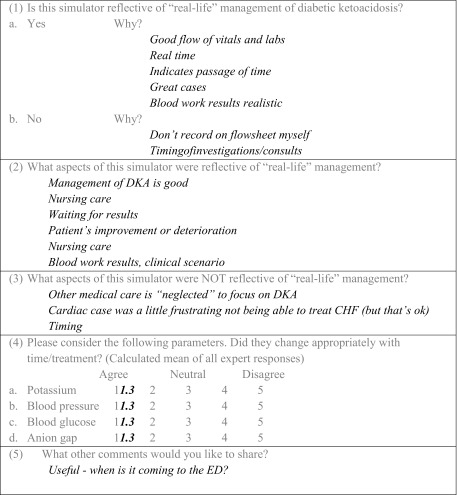
Expert content review

**Figure 3.**
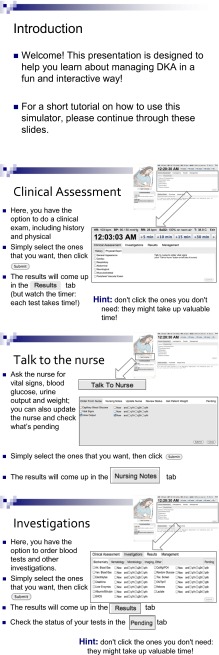

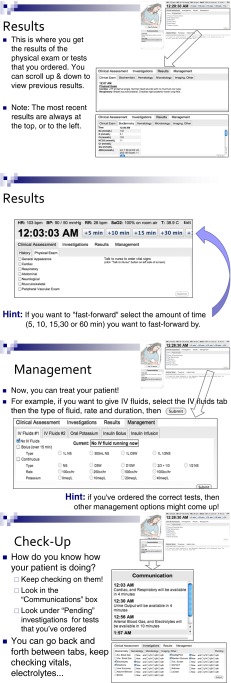

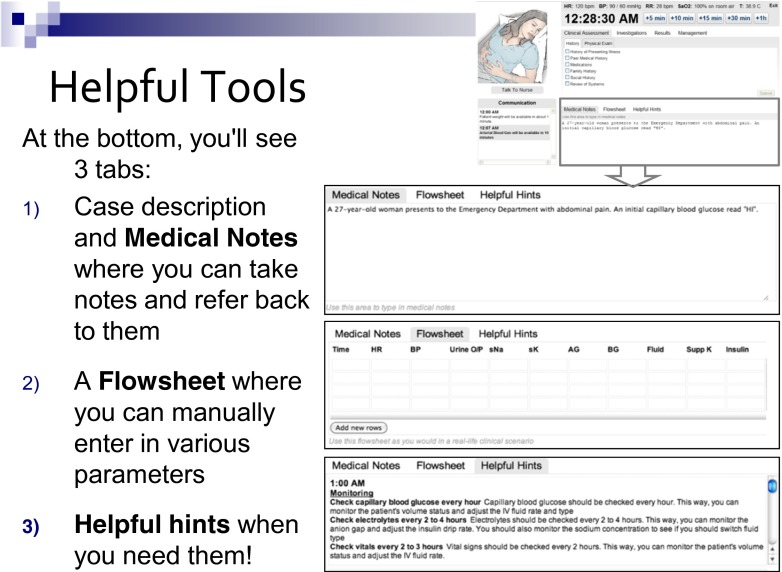
Simulator tutorial.

**Figure 4.**
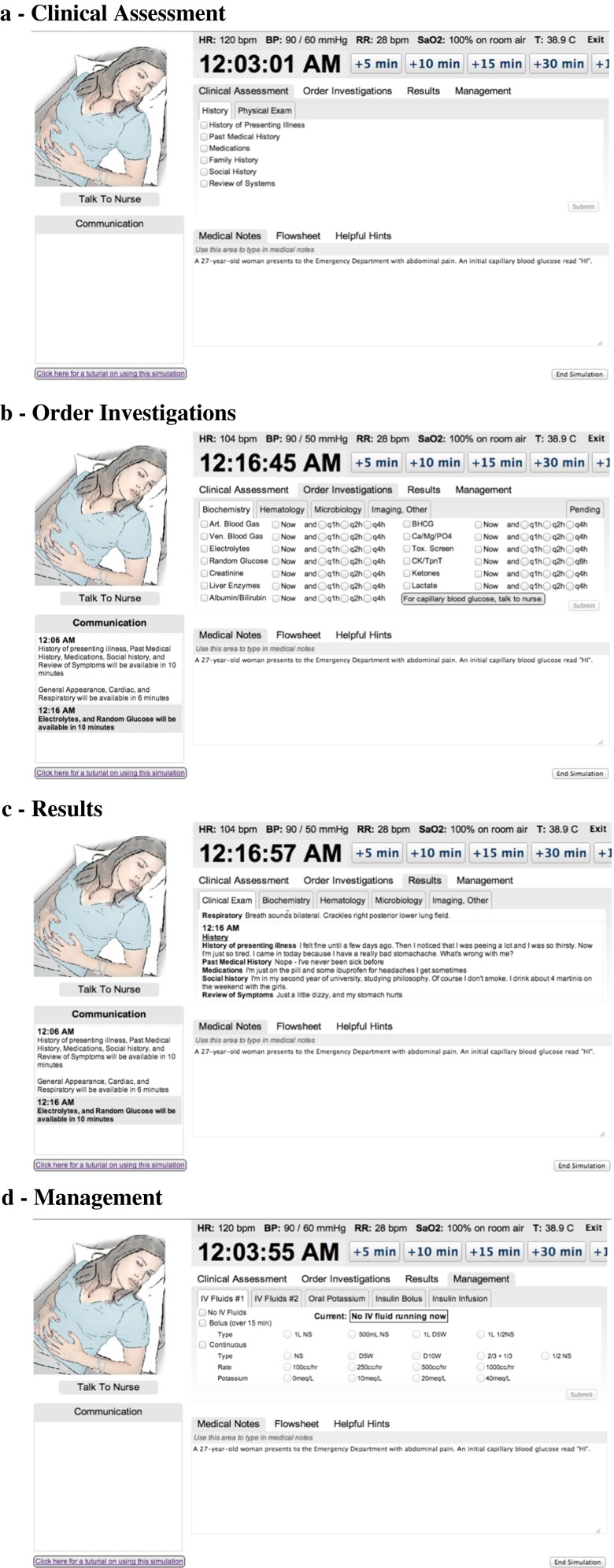

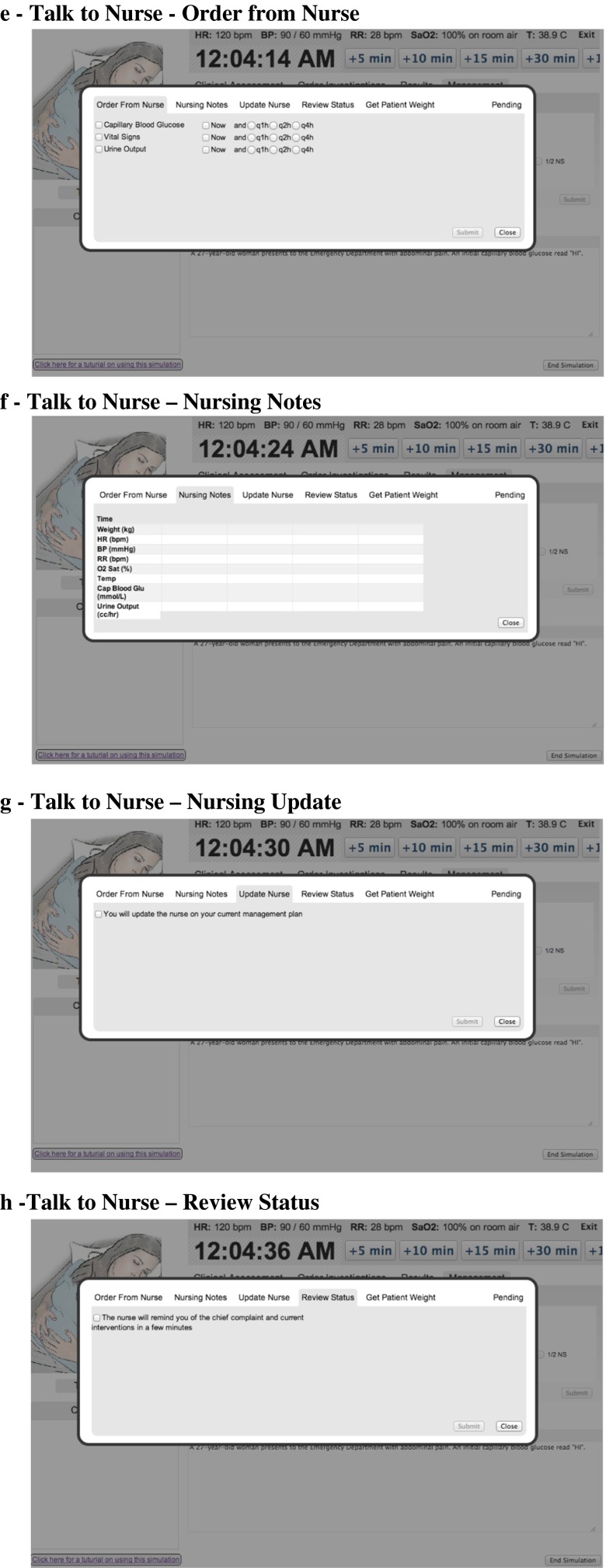

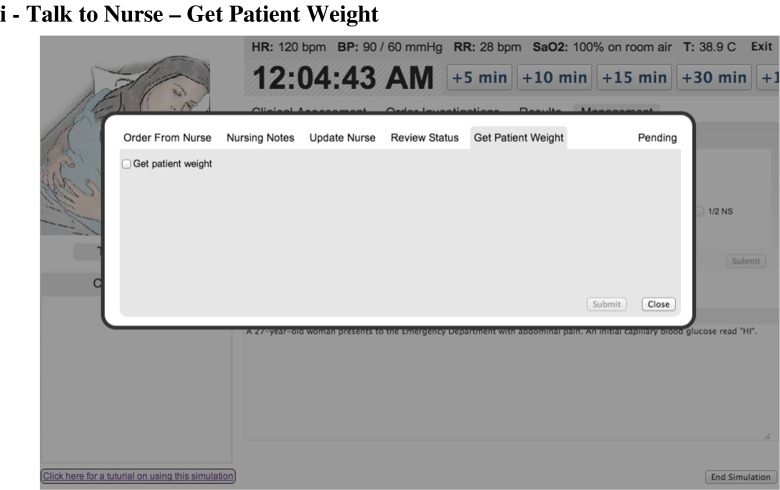
Screen shots of simulator. **a** Clinical assessment. **b** Order investigations. **c** Results. **d** Management. **e** Talk to nurse—order from nurse. **f** Talk to nurse—nursing notes. **g** Talk to nurse—nursing update. **h** Talk to nurse—review status. **i** Talk to nurse—get patient weight.

## Appendix 1

**Table 4.** List of clinical scenarios

Case title	Feature	Case brief	Precipitating cause
Doctor, I have a stomach ache	Classic presentation	A 21-year-old female presents to the Emergency Department with abdominal pain. She has a history of diabetes. An initial capillary blood glucose read “HI”	Pneumonia
Hey Doc, I’ve been getting this pain in my chest	Has chronic renal failure, develops CHF	A 55-year-old male presents to the Emergency Department with chest pain. He has had diabetes for 20 years	Acute coronary syndrome
I don’t know why I’m here… Can I go yet?	Resistant hypokalemia	An 18-year-old female is brought by her friend to the Emergency Department. She wants to leave	Insulin omission due to anorexia nervosa
I’m so thirsty—can I have some water?	Diagnosis	A 27-year-old male is sent in from his family doctor’s office for abnormal blood work	New onset type 1 diabetes
What’s wrong with my dad?	HHS	A 73-year-old male is brought in from his retirement home by emergency medical personnel for altered level of consciousness	Urinary tract infection
So…short…of breath…	Concurrent respiratory acidosis	A 32-year-old female comes in short of breath	Asthma

## Appendix 3

**Table 6.** Scoring system

List of performance items		
Priorities of DKA management	Performance item	
(1) Potassium deficiency	i. Potassium checked prior to initiation of therapy	
	ii. Potassium replaced	
(2) Volume depletion and fluid replacement	iii. Volume status assessed	
	iv. Appropriate fluid selected	
	v. Appropriate rate selected	
	vi. At least 3 l of fluid given by first 4 h	
(3) Acidosis	vii. Acid-base status assessed	
	viii. Insulin therapy selected	
	ix. Appropriate dose selected	
(4) Hyperglycemia	x. Appropriate fluid selected	
	xi. Appropriate rate selected	
(5) Precipitating cause	xii. Investigations ordered	
	xiii. Treatments ordered	
(6) Organization of care	xiv. Communication with nurse	
	xv. Use of flowsheet	
(7) Monitoring	xvi. Capillary blood glucose checked every hour	
	xvii. Electrolytes checked every 2 to 4 h	
	xviii. Vitals checked every 2 to 3 h	
Three-point scoring scale		
Number of points	Level of performance	Description
1 point	Unacceptable	Correct decision or treatment made <50 % of the time*, or critical error** committed
2 points	Borderline performance	Correct decision or treatment made 50–80 % of the time, no critical errors*
3 points	Acceptable	Correct decision or treatment made >80 % of the time, no critical errors*
Critical errors		
Domain		Critical error
(1) Potassium deficiency		Not treating with K
		Initiation of insulin when potassium is less than 3.3
(2) Volume depletion and fluid replacement		Not treating with fluid
(3) Acidosis		Not treating with insulin
		Reduction in insulin for blood glucose less than 14.0 mmol/l (252 mg/dl) with persistent elevated anion gap
(4) Hyperglycemia		Not treating with fluid
(5) Precipitating cause		Not ordering investigations for precipitating cause
(6) Organization of care		None
(7) Monitoring		Blood work frequency > q6h

The six priorities in DKA management make up the six domains of the scoring system, which are (1) potassium deficiency, (2) volume depletion and fluid replacement, (3) acidosis, (4) hyperglycemia, (5) precipitating cause, and (6) organization of care (e.g., communication with nurse, use of a flowsheet, monitoring of patient). This comprises a total of 18 performance items, as outlined below. A three-point scoring scale for each performance item was used as outlined in below. Actions committed throughout the scenario are be tabulated by the simulator into a final numerical score, ranging from 18 to 54, where 15 represents unacceptable performance in all performance items and 54 represents acceptable performance in all performance items

*Because of the iterative nature of DKA management, multiple decisions regarding the same performance item will be made for each case; assessment will be based on all decisions made, as outlined in this table (i.e., if fluids were selected correctly 4 out of 6 times, the learner would receive a score of 2 points for that performance item).

**Examples of critical errors include: initiation of insulin when potassium is less than 3.3 and reduction in insulin for blood glucose less than 14.0 mmol/l (252 mg/dl) with a persistent elevated anion gap
